# Barriers to Clinician Implementation of Parent-Child Interaction Therapy (PCIT) in New Zealand and Australia: What Role for Time-Out?

**DOI:** 10.3390/ijerph182413116

**Published:** 2021-12-12

**Authors:** Melanie J. Woodfield, Tania Cargo, Sally N. Merry, Sarah E. Hetrick

**Affiliations:** 1 The Werry Centre, Department of Psychological Medicine, University of Auckland, Auckland 1023, New Zealand; T.Cargo@auckland.ac.nz (T.C.); s.merry@auckland.ac.nz (S.N.M.); s.hetrick@auckland.ac.nz (S.E.H.); 2 Auckland District Health Board, Auckland 1023, New Zealand; 3 Department of Psychology, University of Auckland, Auckland 1023, New Zealand; 4 Centre for Youth Mental Health, University of Melbourne, Parkville, VIC 3010, Australia

**Keywords:** PCIT, Parent-Child Interaction Therapy, parent training, implementation, barriers, facilitators, determinants, time out, time-out

## Abstract

Background: Parent-Child Interaction Therapy (PCIT) is an effective parent training approach for a commonly occurring and disabling condition, namely conduct problems in young children. Yet, despite ongoing efforts to train clinicians in PCIT, the intervention is not widely available in New Zealand and Australia. Methods: We undertook a cross-sectional online survey of clinicians in New Zealand and Australia who had completed at least the 40-h initial PCIT training, to understand the barriers they encountered in their implementation efforts, and the extent to which attitudes toward time-out influenced implementation. The overall response rate was 47.5% (NZ: 60%; Australia: 31.4%). Results: Responses suggested that participants generally viewed PCIT as both acceptable and effective. Australian participants reported seeing significantly more clients for PCIT per week than those in NZ (Medians 0 and 2, respectively; χ^2^(1) = 14.08, *p* < 0.001) and tended to view PCIT as more effective in treating disruptive and oppositional behaviour (95% CI: −0.70, −0.13, *p* = 0.005). Participants currently seeing PCIT clients described it as more enjoyable to implement than those not using PCIT (95% CI: −0.85, −0.10, *p* = 0.01). Thirty-eight percent of participants indicated that they adapt or tailor the standardised protocol, primarily by adding in content relating to emotion regulation, and removing content relating to time-out. Participants generally felt that they had fewer skills, less knowledge, and less confidence relating to the Parent-Directed Interaction phase of PCIT (which involves time-out), compared with the Child-Directed Interaction phase. Conclusion: While we had hypothesised that time-out represented an intra-intervention component that detracted from implementation success, results suggested that clinician concern over the use of time-out was present but not prominent. Rather, the lack of access to suitable equipment (i.e., one-way mirror and ear-piece) and difficulties associated with clients attending clinic-based sessions were barriers most commonly reported by clinicians. We suggest that future research might consider whether and how PCIT might be “re-implemented” by already-trained clinicians, moving beyond simply training more clinicians in the approach.

## 1. Lay Summary

Internationally, behavioural difficulties are among the most common reasons children and families seek help from mental health services. Evidence-based treatments for childhood behavioural difficulties are some of the more effective psychological treatments we have available. Parent-Child Interaction Therapy (PCIT) is an example of an effective treatment, which involves live coaching of a parent with their child from a clinician behind a one-way mirror using an ear-piece for the parent. However, for complex reasons, relatively few providers routinely deliver PCIT. This study surveyed clinicians who had received training in PCIT in New Zealand and Australia, to understand the reasons why they may not be using it as often as they could. We found that while clinicians generally liked PCIT and saw it as effective, lacking suitable equipment, such as a one-way mirror and ear-piece, was a common barrier to their use of PCIT. Exploring how to better implement an existing evidence-based treatment for childhood conduct problems will make PCIT, and other similar treatments, more available to families in New Zealand.

## 2. Background

Internationally, childhood conduct problems represent one of the most common mental disorders diagnosed in children under seven years [1] and one of the most frequent reasons for young children to be referred to mental health services [2]. Parent training for childhood conduct problems is a treatment approach that has a more extensive evidence base than any other psychosocial treatment for any presentation in the child mental health context [3].

### 2.1. Parent-Child Interaction Therapy (PCIT)

Prominent examples of evidence-based manualised (i.e., supported by a session-by-session protocol) parent training programmes available in New Zealand and Australia are The Incredible Years [4], Triple P [5], and Parent-Child Interaction Therapy (PCIT) [6]. PCIT is distinctive in its use of in vivo coaching of a parent with their 2- to 7-year-old child, typically utilising a one-way mirror and discrete ear-piece for the parent [7]. It involves two phases. Child-Directed Interaction (CDI) has a focus on enhancing the parent-child relationship, while Parent-Directed Interaction (PDI) involves teaching parents effective developmentally appropriate discipline techniques including time-out [7]. Meta-analyses of international randomised controlled trials have suggested that PCIT is effective in reducing conduct problems in children, improving child compliance, reducing parent stress, and improving parent emotion regulation and reflective functioning (i.e., the parent’s ability to hold their child’s mental states in mind, when understanding their child’s behaviour) [8,9,10,11]. To become accredited in PCIT with PCIT International (www.PCIT.org, accessed on 10 December 2021), a registered Masters-level clinician (e.g., psychologist, psychiatrist, social worker, therapist) must initially undertake a 40-h training course, followed by fortnightly PCIT supervision and observations of practice until at least two PCIT cases have been completed successfully. This comprehensive training process, which includes role-plays, video reviews, and group discussions, is seen as necessary for the successful acquisition and maintenance of core PCIT skills [12].

### 2.2. PCIT in New Zealand

The first New Zealand PCIT training was held in 2010, and approximately 135 clinicians have been trained in PCIT to date. Direct training costs are approximately $NZD2500 per person. A 2019 anonymous online pilot survey of 84 PCIT-trained clinicians in New Zealand (response rate: 67%) suggested that between 24% and 45% of PCIT-trained clinicians in New Zealand were using PCIT in their practice, and for those who were using PCIT, it constituted a small proportion of their clinical work: an average of 2.4 clients per week, often co-worked with another clinician, so potentially ‘counted twice’ [13]. As such, PCIT was available to only a modest number of families in New Zealand at the time.

Responses suggested that clinicians felt positively towards PCIT, believing their training experience to have been worthwhile, and PCIT to be both an appropriate and effective treatment for childhood conduct problems [13]. However, many clinicians described having encountered barriers to PCIT use, which were broadly described as lacking support from colleagues, managers, or supervisors; limited availability of necessary equipment; and clients having difficulty accessing clinic-based sessions. Some clinicians described a perception that PCIT was intensive and demanding to deliver, and a sense of professional isolation. Clinicians also described a concern that PCIT was detrimentally impactful on colleagues in terms of the high demand for limited clinic space, and a concern that noise from young PCIT clients may disrupt colleagues. Characteristics of the therapy, most often the use of the time-out strategy, were also described as a barrier, with reports of poor acceptability to colleagues, clients, and to some clinicians themselves. These findings supported and extended earlier qualitative research into clinician-reported barriers to PCIT’s use in the United States of America and the Netherlands [14,15].

### 2.3. Implementation Considerations

This pilot survey confirmed that even when there is a compelling evidence base for a treatment that addresses a common and/or debilitating condition, this does not guarantee its place in a therapist’s treatment repertoire [16]. As Kazdin [17] (p. 201) observed, providing training alone is insufficient, and often based on a premise that “once everyone sees our wonderful evidence, they will swarm our laboratories, seize our manuals, and rush back to their clinics, where they will implement our interventions with abandon”. Rather, closing this evidence to practice gap requires purposeful efforts to address barriers to implementing evidence into practice that are encountered along the way [18]. These implementation barriers can occur at any of several inter-related levels or contexts, including (1) the evidence-based treatment (EBT)/innovation itself (feasibility, acceptability, relative advantage, usability), (2) the professional charged with implementing the EBT (attitudes, knowledge, willingness, and motivation to change existing practice), (3) the patient, or recipient of the EBT (attitudes, knowledge, compliance with treatment), (4) the social context (colleagues’ perspectives and opinions, collaborations), (5) the organisational context (resourcing, structures, staffing), and (6) the economic and political context (policies, funding) [19]. It is now widely accepted that an intervention’s context exerts an active and dynamic influence on implementation success [20,21,22].

In practice, it is often not feasible to simultaneously address barriers across all of these complex contexts. It makes good sense to prioritise clinician-level determinants (i.e., barriers and facilitators) as individual behaviour change is critical to implementation success [23]. Individual factors that impact on behaviour change (e.g., attitudes) may be significantly more influential on implementation success than organisation-level determinants, and often represent a target that is more amenable to change [23].

In addition, intra-intervention characteristics may be important given the influence of characteristics of the treatment components themselves, such as their usability or acceptability, on the individual charged with implementation [24]. This includes components that are fixed features of psychological treatments, which have received relatively little attention in the exploration of barriers to implementation [25]. One component of the Parent-Directed Interaction phase of PCIT involves teaching and coaching parents in the use of time-out with their child. Within PCIT, time-out involves a brief and pre-planned period of withdrawal of the parent’s attention, along with restricted access to toys and other desired items, in response to the child’s non-compliance or defiance [7]. It is one component of a collection of behaviour management strategies, which are only utilized after the parent-child relationship has been strengthened in the initial Child-Directed Interaction phase [7]. The indications for time-out, and contraindications, are discussed further in our companion paper in this special issue (see Woodfield, Brodd & Hetrick, in press). Time-out is a technique that is perhaps the most well-studied component of parent training programmes, and there is no empirical evidence to suggest it is harmful [26]. However, recent years have seen growth in public concern around the safety and appropriateness of time-out [27], fuelled by online material and popular press publications. Little is known about clinician attitudes toward time-out, and whether and how these may influence clinicians’ implementation of parent training programmes. These are valid considerations, as research has highlighted, for example, that exposure-based tasks within Cognitive Behavioural Therapy for anxiety disorders are used relatively infrequently by clinicians, and it has been suggested that clinicians’ “negative beliefs about exposure therapy (e.g., that it is unethical, intolerable and unsafe) impede the utilization of this treatment, even among therapists trained to administer it” [28] (p. 364).

### 2.4. Cultural Considerations

In New Zealand, Indigenous Māori tamariki (children) and whānau (families) are disproportionately represented in conduct problems prevalence statistics. The Christchurch longitudinal survey found that individuals with higher levels of conduct problems were more likely to be male, and of Māori ethnicity [29]. Complex factors are likely to contribute to the disproportionate representation of Māori tamariki and whānau in prevalence data, including the negative experiences associated with colonisation (see [30,31,32]). Rather than a psychiatric disorder, childhood conduct problems for Māori ought to be viewed in the context of sociological factors, the political environment, and other complex considerations [30]. Put simply, a Māori view suggests that conduct problems are an “expression of things gone wrong in the child or young person’s world” [30] (p. 20). In Australia, Aboriginal families (including Australian Aboriginal and Torres Strait Islander peoples) have also experienced cultural disconnection, intergenerational trauma, and high levels of disadvantage [33]. It is important to acknowledge the complexity inherent in a culture-informed understanding of the aetiology, diagnosis, and treatment of childhood conduct problems within Indigenous populations, and the associated limitations of a study such as this.

### 2.5. Aims

Our aims were to (1) primarily, more comprehensively and specifically understand the barriers encountered by PCIT-trained clinicians in their attempts to implement the therapy after their training; (2) understand the extent to which time-out represents a barrier to implementation of PCIT; and (3) determine whether PCIT was generally acceptable to clinicians who had received training in the approach, and whether this acceptability differed between PCIT-trained clinicians in New Zealand and Australia. Little is known about the implementation of PCIT within clinical services in Australia and, given the overlapping implementation contexts in Australia and New Zealand, we were interested in understanding similarities and differences. Finally, (4) given that PCIT was developed in the USA, we were also interested in clinicians’ perceptions of the degree of fit between PCIT and their own culture, and their perception of the fit with their clients’ culture.

Fundamentally, we aimed to extend and deepen the existing pilot research, in order to comprehensively canvas the barriers and facilitators described by PCIT-trained clinicians, understand whether there was a difference in these determinants between New Zealand and Australia, and, importantly, the extent to which these impact on implementation success.

## 3. Materials and Methods

### 3.1. Study Design

A cross-sectional online survey of PCIT-trained clinicians’ beliefs, attitudes, and self-reported behaviour, was created and delivered via Qualtrics, which was selected for its security and functionality.

### 3.2. Participants and Setting

Participants were clinicians who had undertaken the 40-h initial PCIT training or its equivalent and were located in New Zealand (NZ) or Australia at the time of the study. Australia was considered sufficiently similar, in terms of the PCIT implementation context (i.e., professional training pathways and employment contexts), to be included in the sample.

A total of 76 responses were received: 54 of these responses were from NZ, where we estimate that the invitation reached 90 of approximately 135 PCIT-trained clinicians, for a response rate of 60%. In relation to the NZ sample, contact details were unavailable for those clinicians who had changed employment since their training (which may have occurred over 10 years previously), and attempts to identify a current email address relied upon authors’ knowledge of the clinician’s current employment context, or an online search of publicly available information. Twenty-two responses were received from Australian clinicians, where we estimate that the invitation reached 70 PCIT-trained clinicians (S. Morgan, personal communication, 26 July 2021), for a response rate of 31.4%.

Participant demographic characteristics are displayed in Table 1.

### 3.3. Materials

The main survey is available in the Supplementary Materials (File S1). A separate survey was created to capture contact details for those who chose to provide their details to enter a draw for one of four $NZD50 department store vouchers. This ensured that contact details could not be correlated with survey responses, to provide anonymity.

Survey items were informed by the earlier pilot study [13] and relevant research literature. As in the previous survey, we continued to include Likert items drawn from the ‘Clinician Use of and Satisfaction with PCIT’ (CUSP) interview schedule [14,15,34]. We added the final three Likert items (“Fits with my own cultural beliefs about parenting”; “Fits with my clients’ cultural beliefs about parenting”; “Can be adapted to be more culturally applicable”).

### 3.4. Procedure

Approval for the study was obtained from the Auckland Health Research Ethics Committee (Ref: AH22277). In New Zealand, invitations to participate were emailed by an administrator in June 2021 to members of a database of PCIT trainees held by Whāraurau, a Ministry of Health-funded workforce development agency, who are responsible for arranging training in PCIT in New Zealand. The invitation was also emailed by the first author (MJW) to members of a database compiled for a previous research study by the researchers [13]. Invitations to participate in the survey were emailed to Australian clinicians by Sue Morgan, National PCIT Trainer for Australia. The first author also posted an invitation to participate to the PCIT International email listserve (which predominantly reaches those accredited in providing PCIT) and specified that only those clinicians based in NZ and Australia were eligible to participate. Approximately two weeks later, one reminder email was sent to the same groups, with the survey closing one week after this reminder email.

### 3.5. Analysis

Quantitative data analysis primarily involved the calculation of descriptive statistics. Independent-sample *t*-tests were carried out to test for differences in the mean Likert scale rating for each item between NZ and Australian participants, and between those who reported seeing some PCIT clients and those who reported seeing no PCIT clients, with equal variances not being assumed (Welch *t*-test). Due to statistical testing for multiple responses to statements regarding PCIT, a threshold for statistical significance of *p* < 0.01 was used.

A frequency count was undertaken of qualitative written content obtained from open-response fields (e.g., “Please describe what content of material you tend to leave out (from the manualised protocol)”).

Data relating to three open-ended qualitative questions (“If you have persisted with PCIT, what has sustained you?”, “If you no longer use PCIT, what would have made it easier to continue?”, and “Is there anything else you would like to add?”) will be integrated with qualitative data from a series of clinician focus groups that are currently underway, and not reported here.

## 4. Results

**Figure 1:**
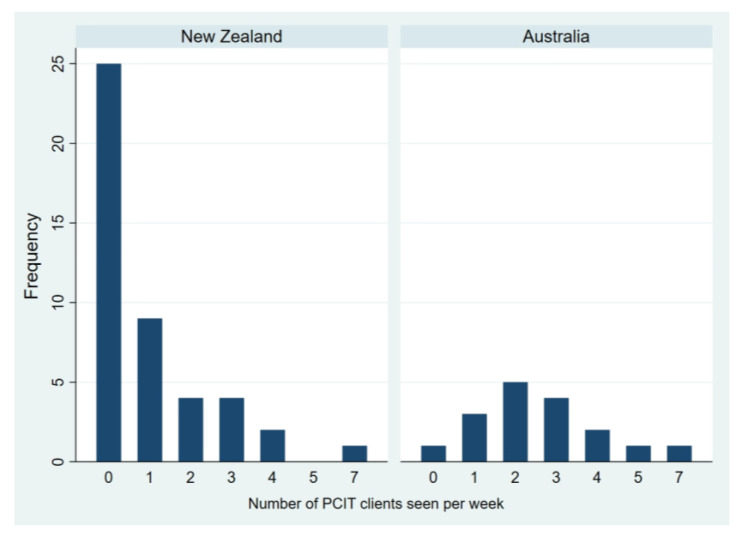
Typical number of PCIT clients/families seen per week.

Of those participants who described currently seeing PCIT clients (53.7% of those responding to this item), the majority described seeing one or two PCIT clients per week in an average or typical week. Results are displayed in Table 2 and represented graphically in Figure 1. In this survey, participants were not required to indicate whether the PCIT sessions were co-worked with another clinician (i.e., potentially ‘counted twice’).

As there was evidence of a non-normal distribution, a nonparametric equality-of-medians test was carried out to determine whether there was a statistically significant difference in the number of PCIT clients seen per week in New Zealand and Australia. The median (IQR) typical number of clients seen for PCIT per week by clinicians was 0 (0 to 1) in New Zealand compared to 2 (2 to 3) in Australia. There was evidence that this difference was statistically significant (χ^2^(1) = 14.08, *p* < 0.001).

### 4.1. Acceptability of PCIT

Table 3 presents the results of the independent-sample *t*-test for differences in the mean Likert scale rating for those participants who reported seeing some PCIT clients, and those who reported seeing no PCIT clients. There was some suggestion that those seeing PCIT clients agreed more with the statements that PCIT “fits with my own cultural beliefs about parenting” and “can be adapted to be more culturally applicable”. However, these differences were not statistically significant at the 1% level (*p ≤* 0.01 for all comparisons). There was evidence of a difference between those seeing PCIT clients and those not seeing PCIT clients in terms of whether they view PCIT as “enjoyable to implement”, with those not seeing PCIT clients being less likely to agree with this statement by an average of 0.47 Likert scale points (95% CI: −0.85, −0.10, *p* = 0.01).

Table 4 presents the independent-sample *t*-test results relating to differences in the mean Likert scale ratings for participants from New Zealand and Australia. NZ participants reported less agreement that PCIT “decreases child disruptive and oppositional behaviours” (an average of 0.41 Likert scale points lower (95% CI: −0.70, −0.13, *p* = 0.005)) and “fits with my own cultural beliefs around parenting” (an average of 0.51 Likert scale points lower (95% CI: −0.87, −0.15, *p* = 0.006) being reported compared to Australian participants. There was also evidence of a difference between NZ and Australian participants in terms of whether they view PCIT as resulting in “increases child disruptive and oppositional behaviours”, with NZ participants being more likely to agree with this statement by an average of 0.48 Likert scale points (95% CI: 0.19, 0.77, *p* = 0.002).

### 4.2. Adaptations to the Manualised PCIT Protocol

Twenty-nine participants (38%) indicated that they adapt or tailor the manualised PCIT protocol in some way. Twenty participants (69%) indicated that they add in content or material, and 13 (45%) remove or leave out content or material. The most common additions to the PCIT protocol related to supporting parents to identify, validate, and support regulation of their child’s emotions, which was referred to in 9 of 20 qualitative comments. An illustrative comment was *“So much happens in a play interaction with capacity for supporting emotion regulation. I think it’s helpful to coach parents around this if it comes up (e.g., ‘it’s okay to reflect back what you think s/he might be feeling’… or ‘it’s okay to give him/her a little rub on the back’, or a labelled praise around staying calm…”*. Other material that participants reported adding in included psycho-education for parents around a child’s experience of trauma; addition of a token economy system; and specific adaptations for children with an Autism Spectrum Disorder, or for younger children (i.e., toddlers).

The most common material to be left out or removed was that relating to the Parent-Directed Interaction phase, specifically time-out for children: “PDI” or “time-out” was mentioned in 10 of the 13 qualitative comments.

### 4.3. CDI and PDI

A higher proportion of participants self-reported having the skills, knowledge, and confidence to both teach and coach CDI compared with PDI (see Table 5).

### 4.4. Barriers and Facilitators to PCIT’s Use

Thirteen (18%) participants reported having not encountered any barriers to using PCIT in their work. Of the participants who had encountered barriers, five (7%) described the barriers they had encountered as predominantly relating to something within PCIT itself, or their feelings about PCIT. Fifty-six (76%) described the barrier as predominantly relating to factors outside of PCIT.

As displayed in Table 6, lacking suitable equipment (i.e., a one-way mirror and ear-piece for the parent) and difficulties for families in attending clinic-based sessions were the most commonly cited barriers for the overall sample. Boxplots displaying the nature of barriers described by the overall sample, and by country, are included in the Supplementary Materials (Figures S1 and S2).

**Figure 2:**
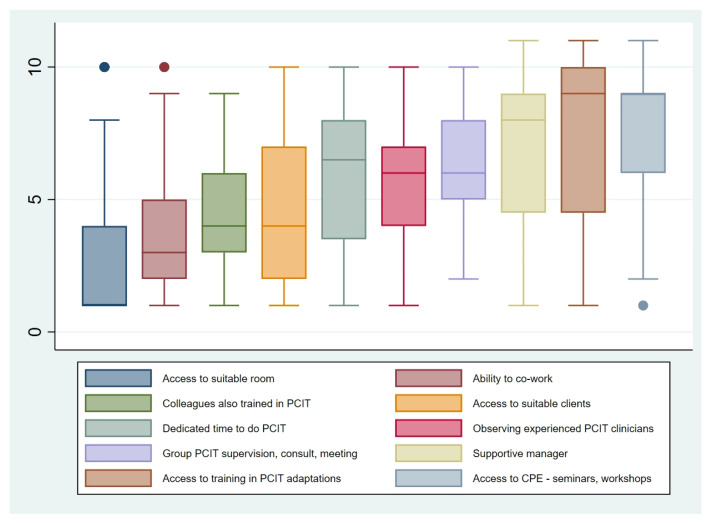
Rank order of facilitators (where 1 = most influential/most significant facilitator) reported by clinicians, overall sample.

**Figure 3:**
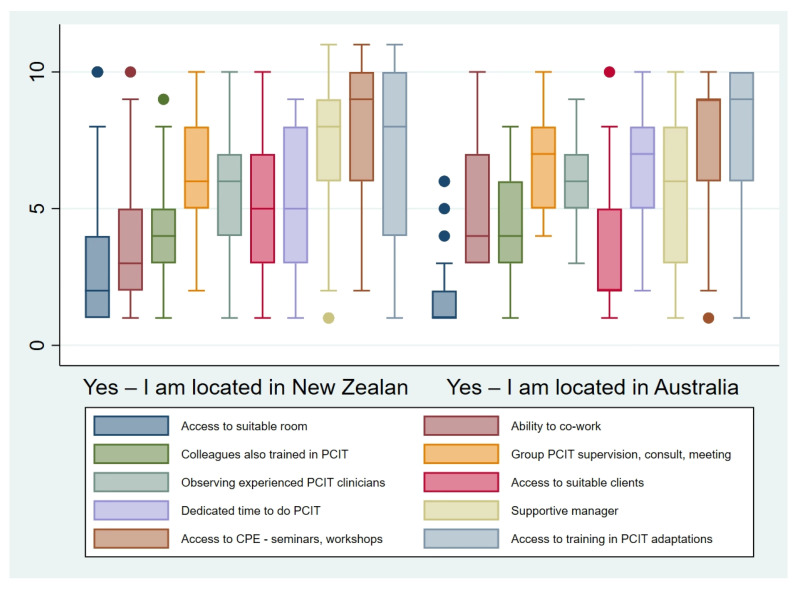
Comparison of rank order facilitators reported by clinicians in New Zealand and Australia.

In keeping with these reported barriers, access to a suitable room was the most commonly cited facilitator to PCIT’s use across New Zealand and Australia. The ability to co-work PCIT cases with another clinician was another commonly reported facilitator, particularly in New Zealand. Figure 2 and Figure 3 display the facilitators reported by the sample as a whole and by country, respectively.

Participants were asked to specify the most helpful contributions of a “supportive manager” if they endorsed this as a facilitator to their use of PCIT. Participants made reference, via brief qualitative responses, to their supportive manager (in order of frequency) ‘ring fencing’ dedicated clinical time for PCIT, providing suitable equipment, arranging or supporting PCIT supervision and training, facilitating a stream of suitable referrals, and engaging in (unspecified) advocacy for PCIT.

### 4.5. Culture

Nineteen participants (26% of those responding to this item) described encountering barriers that related to their clients’ culture. Qualitative comments described an Eastern European family who “*were opposed to using time-out as it did not align with their philosophy of their child being punished for having a “mind of his own”. They dropped out of treatment…*”. One respondent described Aboriginal elder clients as *“not keen on using PCIT, some grandmothers were directly opposed to the use of PDI in particular*”. There was also reference to some families not viewing child-led play as valuable from an (unspecified) cultural viewpoint, or this play being dissonant with an (unspecified) cultural view that the parent ought to be in charge.

The minority (7 participants; 10% of those responding to this item) had experienced barriers that related to their own culture and their use of PCIT. In the associated qualitative comments, three participants within this group referred to difficulties associated with being a non-Māori clinician working with a Māori family.

Responses to the items that related specifically to Māori clinicians’ use of PCIT (i.e., “If you’re Māori, please comment on any barriers you have faced”, “If you’re Māori, please comment on any things that have made using PCIT easier”) have been retained and will be analysed at a later date by an Indigenous Māori-led team, including one of the present authors (TC).

### 4.6. Time-Out within PCIT

**Figure 4:**
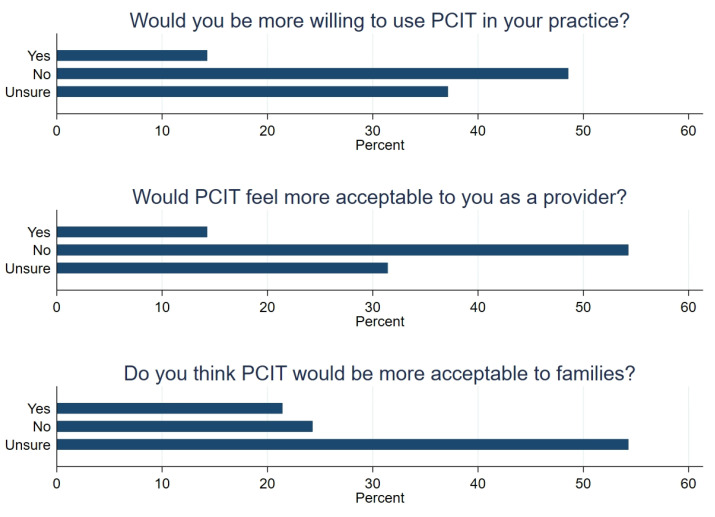
Responses to a hypothetical scenario where time-out was removed from the PCIT protocol.

Participants were asked to consider a hypothetical scenario where time-out was removed from the PCIT manualised protocol, and to indicate whether this would contribute to them (a) being more willing to use PCIT in their practice, (b) PCIT feeling more acceptable to them, and (c) PCIT being more acceptable to families. Results are represented graphically in Figure 4.

The majority (56 participants; 80% of those responding to this item) indicated that they have no, or very few, concerns about time-out within PCIT. Of the minority who did have concerns about time-out, these concerns most commonly related to a view that time-out was unsuitable for traumatised children, and for children with attachment difficulties.

**Figure 5:**
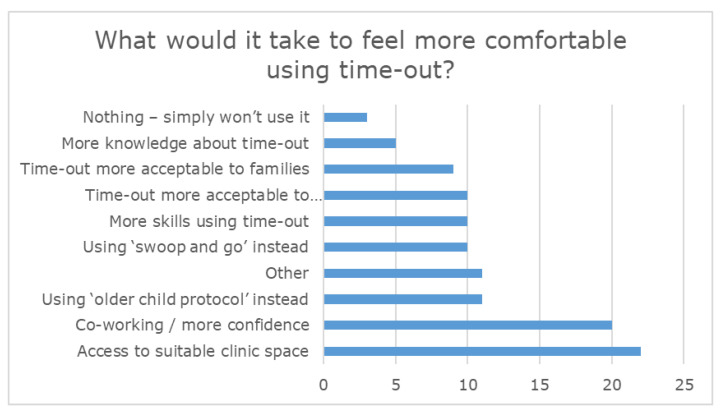
Factors that would assist clinicians to feel more comfortable about using time-out with children.

When asked to comment on what it would take for them to feel more comfortable using time-out, 28 participants indicated that this item was not applicable, as they were already comfortable using time-out. Access to a suitable clinic space (22 participants) and co-working with an experienced PCIT therapist (20 participants) were the most commonly endorsed items by the remaining participants, as illustrated in Figure 5.

## 5. Discussion

This cross-sectional survey of PCIT-trained clinicians in New Zealand and Australia sought to ascertain how acceptable PCIT is to PCIT-trained clinicians, to understand the barriers and facilitators of PCIT’s use, and what—if any—role that intra-intervention components, such as time-out, plays in implementation success. In keeping with an earlier pilot survey, PCIT was broadly viewed as both acceptable and effective by clinicians, with very few differences in acceptability between those who were using PCIT and those who were not. The majority of participants described having encountered barriers to their use of PCIT after their training, and lack of access to suitable equipment, along with clients having difficulty accessing clinic-based sessions featured prominently. Participants were required to indicate whether the barriers were predominantly related to factors ‘outside’ of PCIT, or within PCIT itself, and the majority endorsed the former. This arbitrary dichotomy was intended to assist with determining whether intra-intervention components were key barriers to address to improve implementation. However, some aspects of PCIT may only manifest as a barrier when interacting with a wider system, i.e., outside of PCIT. An applied example includes time-out, which may be acceptable to PCIT clinicians but not to their colleagues or managers. Social influences, such as the views of professional colleagues, can be very relevant to implementation success [35].

Clinicians tended to rate PCIT as less enjoyable to use if they were not using it in their work, possibly due to fewer opportunities to develop a sense of mastery of the components associated with the manualised protocol. New Zealand participants viewed PCIT as less effective in improving children’s behaviour problems but saw fewer PCIT clients per week than their Australian colleagues. Around one-third of participants indicated that they regularly adapt the PCIT protocol, tending to add in content relating to emotion regulation, and commonly removing content relating to time-out. Time-out was not described as a prominent barrier to PCIT’s implementation; however, indirect indicators of the influence of time-out on clinician implementation suggested its role requires further exploration. For example, clinicians described fewer skills, less knowledge, and less confidence teaching and coaching Parent-Directed Interaction (cf. CDI) to parents, and time-out is significant component of PDI. Of interest, when asked to describe what would assist with feeling more comfortable using time-out, participants endorsed having access to suitable clinic rooms, which was the more frequently endorsed barrier generally.

There are a number of factors that might account for the significant difference in the number of clients seen for PCIT per week by Australian and NZ clinicians. The relatively low response rate in Australia might mean that those who did respond were more likely to be clinicians who have an interest in PCIT and/or are using it in their work. Additionally, while Australia has a similar treatment context to NZ, one relevant difference relates to reimbursement for services, with psychologists in Australia often being based in a private practice setting. Reimbursement may be relevant to sustainment of evidence-based practices in particular [36].

Of note, it is possible that while clinicians may be accurate in their description of barriers and the support they need to overcome these barriers and increase their use of evidence-based practices [37], there may be subtle biases in the way they view and report these barriers. For example, clinicians may be biased toward describing external factors, rather than internal factors, such as motivation or effort [38]. This may manifest as a clinician who feels overwhelmed by the anticipated effort of implementing PCIT, citing the lack of a suitable clinic space as a barrier. This can be understood in the context of relevant implementation theory, such as the COM-B model, which suggests that capability and opportunity are necessary conditions for a clinician’s behaviour to occur, and motivation energises and directs clinician behaviour [35]. Practicing a particular behaviour also improves capability, which can increase motivation [39]. In the context of PCIT, for example, it may be that reduced opportunity to utilise the therapy (perhaps by way of lack of access to equipment) detracts from motivation, as capability does not increase. We view our survey data as one component of a “behavioural diagnosis” [40] of what needs to change—within the environment, or within the clinician—in order for the desired behaviour of increased clinician use of PCIT to occur.

## 6. Limitations

There are several limitations to the conclusions that are able to be drawn from a self-report survey of implementation barriers and facilitators. Implementation contexts are dynamic and ever-changing [41]. For example, the funding environment (e.g., for training initiatives), key personnel, or equipment provision may change in the period between this study and any attempt to intervene to address barriers. As such, this study is considered to be a “baseline assessment of context [which will be] used to make key decisions in the next stage of the study” [41] (p. 10).

While a brief deductively driven self-report survey methodology is convenient and low cost, it may not validly capture clinician beliefs and attitudes, and their nuanced interaction with implementation behaviour. For example, organisational factors, such as caseload or time restrictions, might prevent PCIT’s use even when there is high intention to use [42]. Social desirability response bias may also have been a factor [43] despite assurances of anonymity, particularly for NZ participants to whom the research team are known.

The response rate for NZ clinicians was lower than in the pilot survey, which was carried out 2 years earlier. It is possible that NZ clinicians felt that they had already contributed to the research programme. Our research team does not have the same degree of goodwill and professional community in Australia, which may have partly explained the relatively lower response rate there. We were also not able to contact a number of PCIT-trained clinicians in New Zealand, as several had changed roles or were on long-term leave (e.g., parental leave).

It is important to note that this study does not represent, and should not be seen as, a detailed exploration of the acceptability of PCIT to Indigenous practitioners or Indigenous families in New Zealand or Australia. Further research is required to understand the acceptability of PCIT to these groups, and how this influences Indigenous practitioner implementation behaviour. As Māori clinician researcher Emerald Muriwai [44] observed, “we have many modern ‘problems’, which are an echo of our colonized experiences”. Future research into the acceptability of PCIT to Māori ought to be led by Māori, as “Being Māori means that we have already experienced the work we do with our people” [44].

## 7. Future Research

Rather than prioritising the training of more clinicians in PCIT or another parent training approach (though this remains important), we are increasingly interested in better understanding what it would require for already-trained clinicians to begin (or resume) implementing PCIT in their practice. We propose the term “re-implementation”, which we define as *“resuming implementation, implementing again, and/or implementing differently”*. The notion of re-implementation has been described as ‘falling in love again’ with a particular platform after ‘getting started on the wrong foot’ within a computer software context (e.g., https://www.blytheco.com accessed on 11 December 2021). To our knowledge, it has not previously been described within the implementation science literature. Inevitably, there will be a proportion of clinicians who do not use PCIT post-training for compelling reasons, such as transition to a non-clinical role, cessation of practice, or those to whom the approach is not acceptable. Our interest is in those clinicians to whom PCIT is acceptable, who may have encountered barriers, which have overpowered their initial desire to incorporate PCIT in their practice. Our intention is to triangulate these survey findings with qualitative data drawn from focus groups with clinicians and managers, along with future expert consultation, and a systematic review of the existing literature on PCIT implementation, all of which will inform a re-implementation intervention.

## Figures and Tables

**Table 1:** Participant characteristics.

New Zealand Participants’ Ethnicity (Several Participants Endorsed Multiple Ethnicities)		Australian Participants’ Ethnicity (Several Participants Endorsed Multiple Ethnicities)	
New Zealand European	38	Australian	18
Māori	8	Aboriginal	1
Samoan	1	Chinese	1
Indian	1	Other (Caucasian; Mixed ethnicity (White, Asian); New Zealand European/Pakeha; USA)	4
Other (South African (4); Australian; English; European; Korean; Middle Eastern (Kurdish))	12		
**Gender**			
Female			69 (92%)
Male			6 (8%)
**Professional background**			
Clinical Psychologist, Psychologist, or Trainee Psychologist			58 (76%)
Social Worker			7 (9%)
Nurse			3 (4%)
Psychiatrist			3 (4%)
Psychotherapist			1 (1%)
Occupational Therapist			1 (1%)
Counsellor			1 (1%)
Other (Mental health nurse; CBT Infant and Child Therapist)			2 (3%)
**Current employment setting** (several participants endorsed multiple employment contexts)			
Not currently working in a clinical role			1
ICAMHS			23
Private practice			17
Child protection			17
University clinic			9
NGO or charity			7
Education sector			3
Other			10
**Location of clients/families served**			
Mostly urban (e.g., city)			61 (82%)
Mostly rural, remote, or small town			13 (18%)

**Table 2:** Typical number of PCIT clients/families seen per week.

*n* = 67	Count (Percentage)
In a non-clinical role	5 (7.5%)
0	26 (38.8%)
1	12 (17.9%)
2	9 (13.4%)
3	8 (12.0%)
4	4 (6.0%)
5	1 (1.5%)
6	0
7	2 (3.0%)
8 or more	2 (3.0%)

**Table 3:** Difference in Likert scale ratings between those clinicians seeing some PCIT clients, and those not seeing PCIT clients. * indicates statistical significance at the 1% level (*p* ≤ 0.01).

	Participants Who Reported Seeing No PCIT Clients (*n* = 29)			Participants Who Reported Seeing Some PCIT Clients (*n* = 38)					
Overall, I Find PCIT	Mean	SD	Range	Mean	SD	Range	Mean diff.	95% CI	*p*
Easy and straightforward to deliver	4.21	0.90	1–5	4.03	1.13	1–5	0.18	[−0.31, 0.68]	0.47
Helps to keep families in treatment	3.72	0.88	2–5	4.00	0.66	2–5	−0.28	[−0.67, 0.12]	0.16
Decreases child disruptive and oppositional behaviours	4.41	0.73	2–5	4.61	0.82	1–5	−0.19	[−0.57, 0.19]	0.32
Increases family drop-out from treatment	2.66	0.90	1–4	2.45	1.06	1–5	0.21	[−0.27, 0.69]	0.39
Reduces the number of families returning to my agency for additional services	3.17	0.93	1–5	3.26	1.18	1–5	−0.09	[−0.60, 0.42]	0.73
Increases warm and secure interactions between parents and children	4.48	0.87	1–5	4.68	0.47	4–5	−0.20	[−0.56, 0.16]	0.27
Increases child disruptive and oppositional behaviours	1.52	0.74	1–4	1.26	0.55	1–3	0.25	[−0.08, 0.58]	0.13
Lowers parental stress	4.10	0.77	2–5	4.24	0.59	3–5	−0.13	[−0.48, 0.21]	0.44
Enjoyable to implement	4.00	0.85	1–5	4.47	0.60	3–5	−0.47	[−0.85, −0.10]	0.01 *
Complicated and difficult to implement	2.55	1.09	1–4	2.18	1.04	1–4	0.37	[−0.16, 0.89]	0.17
Fits with my own cultural beliefs about parenting	3.97	1.09	1–5	4.42	0.64	3–5	−0.46	[−0.91, −0.00]	0.05
Fits with my clients’ cultural beliefs about parenting	3.41	1.02	1–5	3.74	0.76	2–5	−0.32	[−0.78, 0.13]	0.16
Can be adapted to be more culturally applicable	3.52	0.78	1–5	3.92	0.67	2–5	−0.40	[−0.77, −0.04]	0.03

1 = Strongly disagree, 2 = Somewhat disagree, 3 = Neither agree nor disagree, 4 = Somewhat agree, 5 = Strongly agree.

**Table 4:** Difference in Likert scale ratings between clinicians in New Zealand and Australia. * indicates statistical significance at the 1% level (*p* ≤ 0.01).

	New Zealand Participants (*n* = 52)			Australian Participants (*n* = 22)					
Overall, I Find PCIT	Mean	SD	Range	Mean	SD	Range	Mean Diff.	95% CI	*p*
Easy and straightforward to deliver	4.00	1.07	1–5	4.18	1.01	2–5	−0.18	[−0.71, 0.34]	0.49
Helps to keep families in treatment	3.83	0.76	2–5	3.95	0.72	2–5	−0.13	[−0.50, 0.25]	0.50
Decreases child disruptive and oppositional behaviours	4.40	0.85	1–5	4.82	0.39	4–5	−0.41	[−0.70, −0.13]	0.005 *
Increases family drop-out from treatment	2.75	0.95	1–5	2.14	0.89	1–4	0.61	[0.15, 1.08]	0.011
Reduces the number of families returning to my agency for additional services	3.23	0.94	1–5	3.14	1.32	1–5	0.09	[−0.54, 0.73]	0.76
Increases warm and secure interactions between parents and children	4.52	0.73	1–5	4.77	0.43	4–5	−0.25	[−0.53, 0.02]	0.07
Increases child disruptive and oppositional behaviours	1.62	0.91	1–5	1.14	0.35	1–2	0.48	[0.19, 0.77]	0.002 *
Lowers parental stress	4.10	0.72	2–5	4.32	0.57	3–5	−0.22	[−0.54, 0.09]	0.16
Enjoyable to implement	4.10	0.77	1–5	4.45	0.80	2–5	−0.36	[−0.77, 0.05]	0.08
Complicated and difficult to implement	2.56	1.11	1–5	2.09	1.02	1–4	0.47	[−0.07, 1.00]	0.09
Fits with my own cultural beliefs about parenting	4.08	0.93	1–5	4.59	0.59	3–5	−0.51	[−0.87, −0.15]	0.006 *
Fits with my clients’ cultural beliefs about parenting	3.50	0.90	1–5	3.82	0.80	2–5	−0.32	[−0.74, 0.11]	0.14
Can be adapted to be more culturally applicable	3.81	0.72	2–5	3.64	0.91	1–5	0.17	[−0.27, 0.61]	0.43

1 = Strongly disagree, 2 = Somewhat disagree, 3 = Neither agree nor disagree, 4 = Somewhat agree, 5 = Strongly agree.

**Table 5:** Number (proportion) of participants responding ‘Yes’ to the question “Do you feel you have the [skills, knowledge, confidence] to successfully teach and coach this phase to parents?”.

	Skills	Knowledge	Confidence
**CDI**	74 (98.7%)	72 (96.0%)	68 (90.7%)
**PDI**	65 (90.3%)	66 (91.2%)	50 (69.4%)

**Table 6:** Mean clinician ranking of the influence or significance of barriers to implementation of PCIT post-training.

(*n* = 58)	Mean Ranking (1 = Most Influential/Most Significant Barrier, 15 = Least Influential or Significant)	SD	Range
Lack of access to suitable equipment	4.41	4.34	1–14
Lack of access to suitable clients—unsuitable age range or presenting problems	5.97	3.71	1–15
I feel that my clients’ needs are too severe or complex for PCIT	6.33	4.18	1–14
Families discomfort with/resistance to CDI	6.71	2.90	2–13
Families discomfort with/resistance to PDI	5.95	2.48	1–13
Families unable to easily attend clinic-based sessions (e.g., childcare, transport difficulties)	3.95	2.80	1–14
Families discomfort with being observed (and/or discomfort with video recording, one-way mirror, earpiece)	6.14	2.94	1–14
I lack confidence in delivering PCIT	9.47	2.80	1–14
I lack skills in delivering PCIT	10.41	2.70	3–15
I lack knowledge in delivering PCIT	10.90	2.54	2–15
Lack of support to use PCIT by my manager, team leader or colleagues	8.26	4.33	1–15
Difficulties associated with time out—the practicalities, and/or my feelings about time out	6.98	3.49	1–13
PCIT’s parenting practices do not fit the cultural needs of my clients	9.66	3.53	2–15
PCIT’s parenting practices do not fit with my own cultural beliefs	12.31	2.35	4–15
Other			

## Data Availability

The data presented in this study are available on request from the corresponding author.

## Supplementary Materials

The following are available online at https://www.mdpi.com/article/10.3390/ijerph182413116/s1, Figure S1: Rank order of barriers (where 1 = most influential/most significant facilitator) reported by clinicians, overall sample. Figure S2: Comparison of rank order barriers reported by clinicians in New Zealand and Australia. File S1: Survey items.
