# Supplementary material for: Barriers to Clinician Implementation of Parent-Child Interaction Therapy (PCIT) in New Zealand and Australia: What Role for Time-Out?
Source: Int J Environ Res Public Health. 2021 Dec 12;18(24):13116. doi: 10.3390/ijerph182413116 (PMC8700887; doi:10.3390/ijerph182413116)
Supplement: Supplementary file 1 [file ijerph-18-13116-s001.zip › ijerph-1495152-S1.pdf]

Clinician experiences of implementing Parent-Child Interaction Therapy (PCIT) in New Zealand and Aus

Survey Flow

Block: Default Question Block (1 Question)
Standard: Eligibility (4 Questions)
Standard: Demographics (6 Questions)
Standard: PCIT - Current Use (6 Questions)
Standard: PCIT Generally (2 Questions)
Standard: Barriers (6 Questions)
Standard: Facilitators / Enablers (5 Questions)
Standard: Time out (5 Questions)
Standard: Conclusion (2 Questions)

Branch: New Branch
If
If Thank you so very much for your time! You have completed the survey. Would you like to enter th... Yes Is Selected

EndSurvey: Advanced

Branch: New Branch
If
If Thank you so very much for your time! You have completed the survey. Would you like to enter th... Yes Is Not Selected

EndSurvey:

Page Break

Start of Block: Default Question Block

Q1 Clinician Experiences of Implementing
Parent-Child Interaction Therapy (PCIT)
in New Zealand and Australia Kia ora, Thank you for agreeing to complete our survey – we are aware that you have many demands on your time, so have been careful to ensure that this is as brief as possible. For the most part, comments are optional (though are greatly appreciated), and overall, the survey is likely to take around 8-10 minutes to complete. Even if you trained in PCIT but no longer - or have never - used PCIT in your work, please consider participating. Your views are important to our wider understanding of whether and how PCIT is used by clinicians. Full information about the study, including risks and benefits of participating, is available in this information sheet. At the end of the survey, you can choose to proceed to a separate page to enter the draw for one of four $50 Fishpond vouchers. Thank you again for your time! Dr Melanie Woodfield (Melanie.Woodfield@auckland.ac.nz) (And the research team: Assoc. Prof. Sarah Hetrick, Prof. Sally Merry, and Tania Cargo) *Approved by the Auckland Health Research Ethics Committee on 18/06/2021 for three years. Reference number AH22277.*

O **I consent to participate, and am ready to start the survey** (1)

End of Block: Default Question Block

Start of Block: Eligibility

Q2 **Have you ever completed a five-day initial training (or its equivalent) in Parent-Child Interaction Therapy (PCIT)?**

O Yes (1)

O No (2)

Display This Question:

If Have you ever completed a five-day initial training (or its equivalent) in Parent-Child Interacti... = No

Q4
This survey aims to better understand the challenges PCIT-trained therapists face after their five-day initial training. As such, while we are grateful for your willingness to complete the survey, we have no further questions. Thank you for your time.

Skip To: End of Survey If This survey aims to better understand the challenges PCIT-trained therapists face after their fiv... Is Displayed

Q5 **Are you located in New Zealand or Australia?**

- O Yes – I am located in New Zealand (1)
- O Yes – I am located in Australia (2)
- O No (3)

Display This Question:

If Are you located in New Zealand or Australia? = No

Q6
This survey aims to better understand the challenges New Zealand and Australian PCIT-trained therapists face after their five-day initial training. As such, while we are grateful for your willingness to participate, we have no further questions. Thank you for your time.

Skip To: End of Survey If This survey aims to better understand the challenges New Zealand and Australian PCIT-trained ther... Is Displayed

End of Block: Eligibility

Start of Block: Demographics

Display This Question:

If Are you located in New Zealand or Australia? = Yes – I am located in New Zealand

Q7 **Which ethnic group do you belong to?** *Mark the space or spaces that apply to you*

- ▢ New Zealand European (1)
- ▢ Māori (2)
- ▢ Samoan (3)
- ▢ Cook Island Māori (4)
- ▢ Tongan (5)
- ▢ Niuean (6)
- ▢ Chinese (7)
- ▢ Indian (8)
- ▢ Other (such as Dutch, Japanese, Tokelauan). Please state: (9) ____

Display This Question:

If Are you located in New Zealand or Australia? = Yes – I am located in Australia

Q8 **Which ethnic group do you belong to?***Mark the space or spaces that apply to you*

- ▢ Australian (1)
- ▢ Aboriginal (2)
- ▢ Torres Strait Islander (3)
- ▢ New Zealand Māori (4)
- ▢ Samoan (5)
- ▢ Cook Island Māori (6)
- ▢ Tongan (7)
- ▢ Niuean (8)
- ▢ Chinese (9)
- ▢ Indian (10)
- ▢ Other (such as Dutch, Japanese, Tokelauan). Please state: (11) ____

Q9 **What is your gender?**

- O Prefer not to say (1)
- O Female (2)
- O Male (3)
- O Diverse / nonbinary (4)

Q10 **What is your professional background?** *Please select all that apply.*

- ☐ Clinical Psychologist, Psychologist or Trainee Psychologist (1)
- ☐ Psychotherapist (2)
- ☐ Social Worker (3)
- ☐ Occupational Therapist (4)
- ☐ Nurse (5)
- ☐ Psychiatrist (6)
- ☐ Paediatrician (7)
- ☐ Counsellor (8)
- ☐ Other, please state: (9) ____

Q11 **What type of service / agency do you currently work for?***Please select all that apply.*

▢⊗**I’m not currently working in a clinical role** (1)

▢Infant, Child and Adolescent Mental Health Service (ICAMHS) (2)

▢Private practice (3)

▢Child protection service (e.g. Oranga Tamariki in New Zealand or Department of Communities and Justice in Australia) (4)

▢Non-Government Organisation (NGO) or charity (5)

▢School / Ministry of Education (6)

▢University clinic (7)

▢Other, please state: (8) ____

Q12 **Where are your clients predominantly located?**

O Mostly urban (e.g. city) (1)

O Mostly rural, remote, or small town (2)

O Not applicable (3)

End of Block: Demographics

Start of Block: PCIT - Current Use

Q14 *This section asks about how you use (or used) PCIT in your work. First, we will ask about your use of the full standard protocol, and then about any adaptations you may have made.*

Q16 **How many different PCIT clients do you currently see <u>per week</u>** in an average or typical week**?** *Please note, this only refers to using the <u>full programme</u> according to the PCIT manual.*

For example, if you integrate PCIT concepts into your work but don't adhere to a protocol, you might answer "0".

- O **I am in a non-clinical role** (1)
- O 0 (2)
- O 1 (3)
- O 2 (4)
- O 3 (5)
- O 4 (6)
- O 5 (7)
- O 6 (8)
- O 7 (9)
- O 8 or more (10)
- O Comment (optional): (11) ____

Q17 **Do you / did you adapt or tailor the standard PCIT protocol in any way?** *For example, adding in or removing components, or 'doing things differently' to the standard PCIT protocol*

- O Yes (1)
- O No (2)
- O Not applicable - I have never used PCIT in my work (3)

Display This Question:

If Do you / did you adapt or tailor the standard PCIT protocol in any way? For example, adding in or... = Yes

Q19 *Do you / did you **add in content or material**? For example, augmenting or enhancing the existing PCIT protocol?*

- O No (1)
- O Yes. Please describe what content or material you tend to add in: (2) ____

Display This Question:

If Do you / did you adapt or tailor the standard PCIT protocol in any way? For example, adding in or... = Yes

Q20 *Do you / did you **remove, or leave out content or material**? For example, leaving out aspects of the PCIT protocol you don't see as necessary or effective.*

- O No (1)
- O Yes. Please describe what content or material you tend to leave out: (2) ____

Page Break

Q39 Please consider how you currently feel about each phase of PCIT in relation to the questions in the following table:

If you are not currently using PCIT, please consider how you would feel if you were hypothetically planning to start using it again.

	**CDI – Child Directed Interaction**			**PDI - Parent Directed Interaction**		
	Yes (1)	No (2)	Unsure (3)	Yes (1)	No (2)	Unsure (3)
Do you feel you have the **skills** to successfully teach and coach this phase to parents? (1)	o	o	o	o	o	o
Do you feel you have the **knowledge** to successfully teach and coach this phase to parents? (2)	o	o	o	o	o	o
Do you feel you have the **confidence** to successfully teach and coach this phase to parents? (3)	o	o	o	o	o	o

End of Block: PCIT - Current Use

Start of Block: PCIT Generally

Q22 *In the following sections, if you are not currently using PCIT in your work, or no longer working in a clinical role, please answer in relation to your previous use of PCIT.*

Q23 **Here are a series of ten statements about PCIT. We are interested in your honest thoughts – even if you no longer use PCIT.**

Please indicate whether you strongly disagree, somewhat disagree, neither agree nor disagree, somewhat agree, or strongly agree with the following statements:

	Strongly disagree (1)	Somewhat disagree (2)	Neither agree nor disagree (3)	Somewhat agree (4)	Strongly agree (5)
Easy and straightforward to deliver (1)	o	o	o	o	o
Helps to keep families in treatment (2)	o	o	o	o	o
Decreases child disruptive and oppositional behaviours (i.e. makes the behaviours go down) (3)	o	o	o	o	o
Increases family drop-out from treatment (i.e. makes more families drop out) (4)	o	o	o	o	o
Reduces the number of families returning to my agency for additional services (5)	o	o	o	o	o
Increases warm and secure interactions between parents and children (i.e. more warm interactions) (6)	o	o	o	o	o

Increases child disruptive and oppositional behaviours (makes the behaviour worse) (7)	o	o	o	o	o
Lowers parental stress (8)	o	o	o	o	o
Enjoyable to implement / use (9)	o	o	o	o	o
Complicated and difficult to implement / use (10)	o	o	o	o	o
Fits with my own cultural beliefs about parenting (11)	o	o	o	o	o
Fits with my clients' cultural beliefs about parenting (12)	o	o	o	o	o
Can be adapted to be more culturally applicable (13)	o	o	o	o	o

End of Block: PCIT Generally

Start of Block: Barriers

Q20 *This section relates to any barriers you may have faced in attempting to use PCIT in your work.*

Q21 **Have you encountered any barriers that have influenced your ability or willingness to use PCIT in your work?**

O No (1)

O Yes – these mostly relate(d) to something **within PCIT itself** or **my feelings about PCIT** (2)

O Yes – these mostly relate(d) to **factors outside of PCIT** (e.g. equipment, supervision, training, clients, managers, systems) (3)

Display This Question:

If Have you encountered any barriers that have influenced your ability or willingness to use PCIT in... = Yes – these mostly relate(d) to something <u>within PCIT itself </u>or <u>my feelings about PCIT</u>

Or Have you encountered any barriers that have influenced your ability or willingness to use PCIT in... = Yes – these mostly relate(d) to <u>factors outside of PCIT</u> (e.g. equipment, supervision, training, clients, managers, systems)

Q23 **Please rank (drag and drop) the following in order of how much of a barrier they are (or were) to your use of the full PCIT protocol. In other words, which of these are most likely to 'get in the way' of your intention to use PCIT?** *(1 = most influential / most significant barrier) Tip: Click on a line, and hold down your mouse button. Drag the line to the*

position you feel it should be. Repeat for any other lines that you feel should be in a different ranking/position.

______ Lack of access to suitable equipment (1)
______ Lack of access to suitable clients – unsuitable age range or presenting problems (2)
______ I feel that my clients' needs are too severe or complex for PCIT (3)
______ **Families** discomfort with / resistance to **CDI** (4)
______ **Families** discomfort with / resistance to **PDI** (5)
______ Families unable to easily attend clinic-based sessions (e.g. childcare, transport difficulties) (6)
______ Families discomfort with being observed (and/or discomfort with video recording, one-way mirror, earpiece) (7)
______ I lack **confidence** in delivering PCIT (8)
______ I lack **skills** in delivering PCIT (9)
______ I lack **knowledge** in delivering PCIT (10)
______ Lack of support to use PCIT by my manager, team leader or colleagues (11)
______ Difficulties associated with **time out** – the practicalities, and/or my feelings about time out (12)
______ PCIT's parenting practices do not fit the **cultural needs of my clients** (13)
______ PCIT's parenting practices do not fit with **my own cultural beliefs** (14)
______ Other: (15)

Display This Question:

If Have you encountered any barriers that have influenced your ability or willingness to use PCIT in... = Yes – these mostly relate(d) to something <u>within PCIT itself </u>or <u>my feelings about PCIT</u>

Or Have you encountered any barriers that have influenced your ability or willingness to use PCIT in... = Yes – these mostly relate(d) to <u>factors outside of PCIT</u> (e.g. equipment, supervision, training, clients, managers, systems)

Q24 **Have you faced barriers to your use of PCIT that relate to culture?**

☐ No (1)

☐ Yes – I have faced barriers relating to **my own culture** and my use of PCIT. *Please comment further:* (2) ____

☐ Yes – I have faced barriers relating to **my clients' culture** and the use of PCIT. *Please comment further:* (3) ____

Display This Question:

If Which ethnic group do you belong to? Mark the space or spaces that apply to you = Māori

Or Which ethnic group do you belong to? Mark the space or spaces that apply to you = New Zealand Māori

Q25 **If you are Māori please comment on any barriers you may have faced related to implementing PCIT in your agency.**

____

____

____

____

____

Display This Question:

If Which ethnic group do you belong to? Mark the space or spaces that apply to you = Māori

Or Which ethnic group do you belong to? Mark the space or spaces that apply to you = New Zealand Māori

Q26 **If you are Māori please comment on any things that have directly facilitated your use of PCIT, or made using PCIT easier.**

____

____

____

____

____

End of Block: Barriers

Start of Block: Facilitators / Enablers

Q28 *Just before we ask a series of specific questions about time out within PCIT – **which will be the final section of the survey** – we want to ask about facilitators, or things that may have made it easier to use PCIT in your work.*

Q29 **Please rank (drag and drop) the following in order of how much of a facilitator they are (or were) to your use of the full PCIT protocol. In other words, which of these are most likely to <u>increase your use of PCIT, or make it easier to use PCIT</u>?** *(1 = most influential / most significant) <u>Tip:</u> Click on a line, and hold down your mouse button. Drag the line to the position you feel it should be. Repeat for any other lines that you feel should be in a different ranking/position.*

______ Access to suitable clinic rooms (1)
______ The ability to co-work cases with another clinician if I choose to (2)
______ Having colleagues in my workplace who are also trained in PCIT (3)
______ PCIT interest group meetings, or group supervision/consultation (4)
______ The ability to observe experienced clinicians delivering PCIT (5)
______ Access to suitable clients – age range and presenting problems (6)
______ Dedicated or 'ring fenced' time to do PCIT (7)
______ Supportive manager. The most helpful thing(s) they do is/are: (8)
______ Easy, low-cost access to ongoing professional development in PCIT (seminars, workshops) (9)
______ Access to training in PCIT adaptations for particular groups (e.g. Toddlers, ASD, Selective Mutism) (10)
______ Other: (11)

Q30 **If you have persisted with using PCIT, why have you done so (i.e., what has sustained you in your PCIT work)?**

____

____

____

____

____

Q31 **If you no longer use PCIT – what would have made it easier to continue to use PCIT in your work?**

____

____

____

____

____

Q32 ***Please proceed to the final section on time out.***

End of Block: Facilitators / Enablers

Start of Block: Time out

Q33 ***Well done for making it to the final section, we really appreciate your time!   Here are a series of questions about 'time out' for young children.***

Q34 **Hypothetically, if time out were removed from the PCIT protocol…**

	Yes (1)	No (2)	Unsure (3)
Would you be more willing to use PCIT in your practice? (1)	o	o	o
Would PCIT feel more acceptable to you as a provider? (2)	o	o	o
Do you think PCIT would be more acceptable to families? (3)	o	o	o

Q36 Which of the following statements best describes your attitude towards time out within PCIT?

- o I have some (or serious) concerns about time out within PCIT (1)
- o I have no (or very few) concerns about time out within PCIT (2)

Display This Question:

If Which of the following statements best describes your attitude towards time out within PCIT? = I have some (or serious) concerns about time out within PCIT

Q35 **Please rank (drag and drop) the following in order of how much of a barrier they are to your use of time out.**

(1 = most influential / most significant) Tip: Click on a line, and hold down your mouse button. Drag the line to the position you feel it should be. Repeat for any other lines that you feel should be in a different ranking/position.

______ Concern that it is **ineffective**, or doesn't work (1)
______ Concern that it is not suitable for **children with a trauma history** (2)
______ Lacking the necessary **equipment** (e.g. time out room) (3)
______ Concern about **noise or disruption for colleagues** (4)
______ Concern that it is not suitable for **children with attachment difficulties** (5)
______ Concern that it does not fit the **cultural values of the families** I work with (6)
______ Concern that it does not fit with **my own cultural values** (7)
______ Concern about my ability to manage the **parent's** emotions and/or behaviour (8)
______ Concern about my ability to manage the **child's** emotions and/or behaviour (9)
______ **Families** that I work with don't like it (10)
______ Concern that time out may **traumatise** (or retraumatize) the child (11)
______ Concern that time out addresses the **superficial** symptoms of a behavioural disorder but does not target their root cause (12)
______ Other: (13)

Q38 **What would it take for you to feel more comfortable using time out in your clinical work with parents? What would need to be different?** *Please select all that apply*

- ▢ ⊗ **Not applicable – I am already comfortable using time out** (1)
- ▢ ⊗ **Nothing – I simply do not think I will ever use it** (2)
- ▢ Using ‘swoop and go’ instead of a time out room (3)
- ▢ Using the ‘older child protocol’ (i.e. removal of privileges until time out complete) (4)
- ▢ Understanding more about the research into the safety and effectiveness of time out (i.e. more knowledge) (5)
- ▢ Having a refresher on how to use time out (i.e. more skills) (6)
- ▢ Co-working PCIT cases with an experienced PCIT therapist (i.e. more confidence) (7)
- ▢ Time out being more acceptable to families (8)
- ▢ Time out being more acceptable to (or supported by) colleagues and managers (9)
- ▢ Improved access to suitable clinic space (e.g. sound proofing, suitable time out room) (10)
- ▢ Other: (11) ____

End of Block: Time out

Start of Block: Conclusion

Q39
Finally, is there anything else that you would like to share about your experience of using PCIT in your work?

(Comments optional, though appreciated)

____

____

____

Q40 **Thank you so very much for your time! You have completed the survey.**
Would you like to enter the draw for one of **four $50 Fishpond vouchers**?
Selecting 'Yes' will take you to a separate link, where you can enter your details.

O Yes (1)

O No (2)

End of Block: Conclusion
