# Supplementary material for: Barriers to Clinician Implementation of Parent-Child Interaction Therapy (PCIT) in New Zealand and Australia: What Role for Time-Out?
Source: Int J Environ Res Public Health. 2021 Dec 12;18(24):13116. doi: 10.3390/ijerph182413116 (PMC8700887; doi:10.3390/ijerph182413116)
Supplement: Supplementary file 1 [file ijerph-18-13116-s001.zip › ijerph-1495152-Supplementary Figures.pdf]

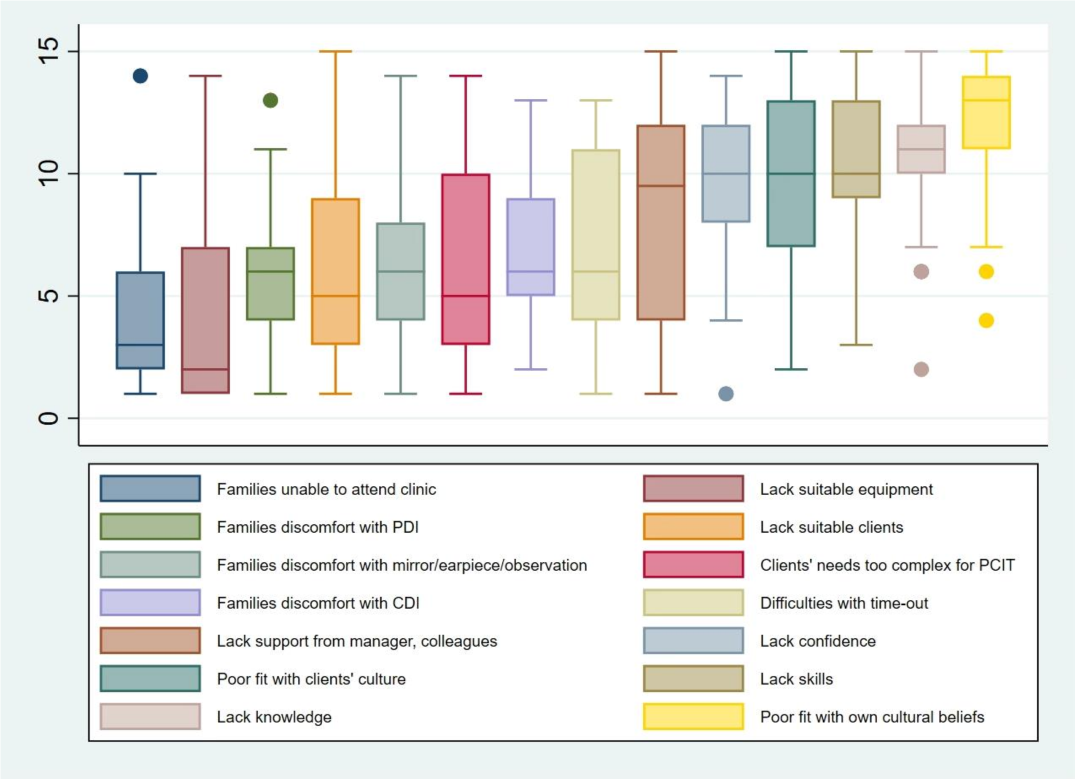

Figure S1. Rank order of barriers (where 1 = most influential / most significant barrier) reported by clinicians, overall sample.

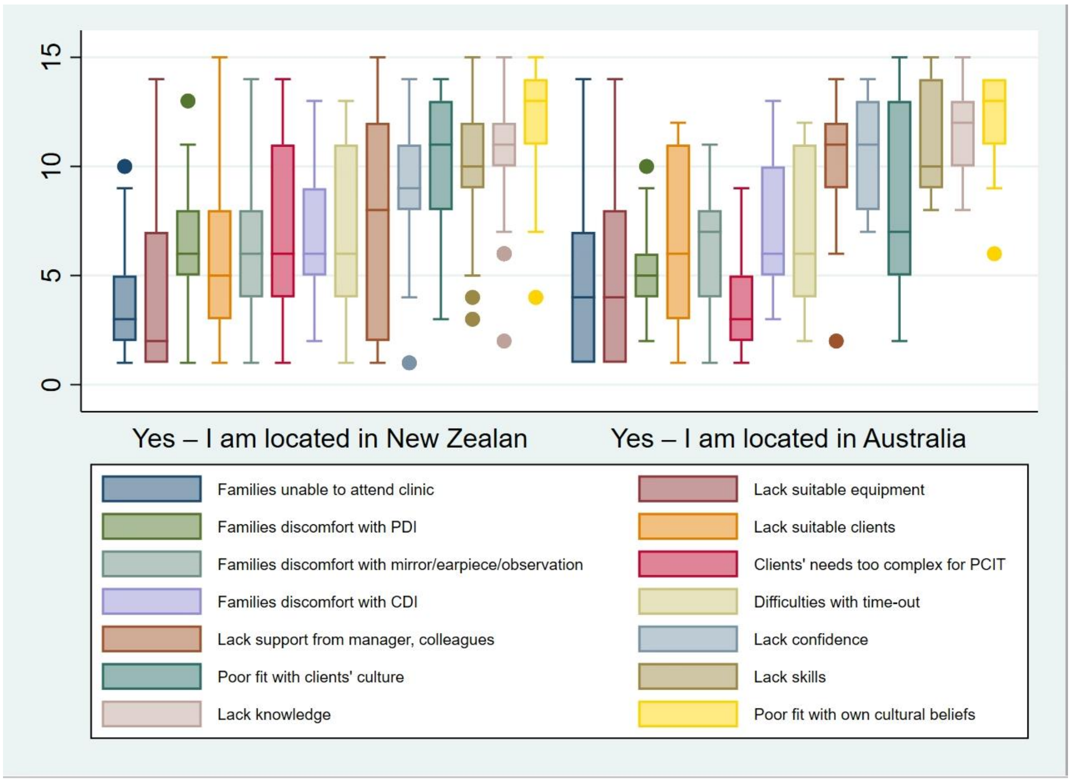

Figure S2. Comparison of rank order barriers reported by clinicians in New Zealand and in Australia.
